# Impact of Nutrient Availability on the Fermentation and Production of Aroma Compounds Under Sequential Inoculation With *M. pulcherrima* and *S. cerevisiae*

**DOI:** 10.3389/fmicb.2020.00305

**Published:** 2020-02-28

**Authors:** Pauline Seguinot, Anne Ortiz-Julien, Carole Camarasa

**Affiliations:** ^1^SPO, INRAE, Univ Montpellier, Montpellier SupAgro, Montpellier, France; ^2^Lallemand S.A.S, Blagnac, France

**Keywords:** sequential inoculation, wine fermentation, nutrient availability, aroma production, fermentation performances, *Saccharomyces cerevisiae*, *Metschnikowia pulcherrima*

## Abstract

Non-*Saccharomyces* yeasts are currently widely used in winemaking to enhance aroma profile diversity among wines. The use of *Metschnikowia pulcherrima* in sequential inoculation with *S. cerevisiae* was compared to the inoculation of a pure culture of *S. cerevisiae*. Moreover, various concentrations of sugar, nitrogen and lipids were tested in synthetic must to assess their impact on fermentation and its outcomes using a Box-Behnken design. Due to its phenotypic specificities, early inoculation with *M. pulcherrima* led to important modifications, first altering the fermentation kinetics. This may relate, at least in part, to the depletion of some nitrogen sources by *M. pulcherrima* during the first part of fermentation. Beyond these negative interactions on fermentation performance, comparisons between pure cultures and sequentially inoculated cultures revealed changes in the distribution of carbon fluxes during fermentation in presence of *M. pulcherrima*, resulting in a positive impact on the production of central carbon metabolites and aromas. Furthermore, the expression of varietal thiols was strongly increased as a consequence of positive interactions between the two species. The mechanism of this release still needs to be investigated. Significant differences in the final concentrations of fermentative and varietal aromas depending on the initial must composition were obtained under both inoculation strategies. Interestingly, the response to changes in nutrient availability varied according to the inoculation modality. In particular, a greater incidence of lipids on the production of fatty acids and their ethyl esters derivatives was found during sequential fermentation compared with pure culture, to be viewed in combination with the metabolic characteristics of *M. pulcherrima* regarding the production of volatile compounds from acetyl-CoA. Overall, the importance of managing nutrient availability under *M. pulcherrima*/*S. cerevisiae* sequential inoculation in order to derive the maximum benefit from the potentialities of the non-*Saccharomyces* species while carrying out fermentation to dryness was highlighted.

## Introduction

After being considered spoilage yeasts for decades, the potential of non-*Saccharomyces* (NS) species to increase the aromatic complexity and improve the sensory properties of wines has been acknowledged, and some NS strains are already available in the market as active dried yeasts. NS strains have been successfully used to decrease the final content of ethanol in wines (Contreras et al., 2014; Varela et al., 2016; Brou et al., 2018) or for the diversification of the aromatic profile of the product, increasing both the formation of fermentative aromas and the release of varietal aromas due to their capacity to excrete hydrolytic enzymes (Egli et al., 2002; Rodríguez et al., 2010; Zott et al., 2011; Belda et al., 2017). Among these compounds, thiols play a central role, particularly in white wines, as they possess sought-after aromas of box tree, citrus and passion fruit. These molecules include 3-mercaptohexan-1-ol (3MH), 3-mercaptohexylacetate (3MHA), and 4-mercapto-4-methylpentan-2-one (4MMP). Regarding their formation, 3MH and 4MMP are released during fermentation by the action of hydrolytic enzymes, the β-lyases, on non-odorous precursors present in grape must (Roncoroni et al., 2011). Glutathione and cysteine S-conjugates as well as cysteinyl-glycine S-conjugates and γ-glutamyl-cysteine *S*-conjugates have been identified as thiol precursors in various grape varieties. Furthermore, 3MH may be formed by sulfur addition to E-(2)-hexenal, but these pathways account for a limited part of total thiol formation. 3MHA is synthetized by the acetylation of 3MH by Atf1p (Roland et al., 2011).

Among the NS species studied, *Metschnikowia pulcherrima* stands out because of presenting properties of technological interest. Despite its weak fermentation ability (Sadineni et al., 2012; Contreras et al., 2014), this species possesses high glycosidase activity, enabling the release of norisoprenoids and terpenes from their glycosylated precursors (Rodríguez et al., 2010), as well as extracellular protease activity that can reduce protein haze formation and increase the liberation of nitrogen sources (Reid et al., 2012). In some strains, β-lyase activity involved in the release of varietal thiols has also been identified (Zott et al., 2011). Furthermore, adding *M. pulcherrima* before *S. cerevisiae* substantially changes the profile of fermentative compounds and aromas produced during winemaking. During *M. pulcherrima*/*S. cerevisiae* fermentation, a higher concentration of glycerol and a lower concentration of acetate have been obtained (Comitini et al., 2011; Sadoudi et al., 2012; González-Royo et al., 2015). This species has also been associated with a reduction in the final content of ethanol (Contreras et al., 2014; Quirós et al., 2014; Varela et al., 2016). Finally, combining *M. pulcherrima* with *S. cerevisiae* results in changes in the formation of aromas, with an increase in the final concentration of higher alcohols (Rodríguez et al., 2010; Sadoudi et al., 2012) and variations in the production of ethyl esters and acetate esters depending on the fermentation conditions (Rodríguez et al., 2010; Comitini et al., 2011; Varela et al., 2016; Hranilovic et al., 2018). Overall, wines fermented with *M. pulcherrima* are perceived as more floral, with smoky aromas (González-Royo et al., 2015).

The key role of nutrient availability in fermentation performance and the formation of volatile compounds has been reported in pure cultures of *S. cerevisiae*. The initial concentration of sugar defines the final amount of ethanol and the fermentation duration, in addition to affecting yeast growth (D’Amato et al., 2006; Ribereau-Gayon et al., 2006; Arroyo-López et al., 2009). Lipid deficiency can lead to stuck or sluggish fermentation because of a reduction in yeast viability (Sablayrolles et al., 1996; Julien et al., 2000). Under oxygen limitation conditions, yeasts are unable to synthesize sterols and fatty acids in the absence of extracellular lipids (Andreasen and Stier, 1953, 1954). In contrast, high concentrations of lipids negatively impact the production of ethyl esters by *S. cerevisiae* (Saerens et al., 2008; Rollero et al., 2014).

Because it is both the limiting factor for yeast growth in most fermentations and a parameter modulating the formation of aroma, the impact of nitrogen availability and composition has been intensively studied. Nitrogen deficiency in grape juice often results in stuck or sluggish fermentation (Bely et al., 1990). Regarding the formation of volatile molecules, in pure *S. cerevisiae* cultures, nitrogen has a quadratic impact on the production of higher alcohols and a positive effect on that of ethyl and acetate esters (Beltran et al., 2005; Jiménez-Martí et al., 2007; Mouret et al., 2014; Rollero et al., 2017). Nitrogen availability during fermentation has an impact on the release of varietal thiols, but the effect is not well characterized. Thus, opposite effects of nitrogen addition (as ammonium) have been reported, either increasing (Harsch and Gardner, 2013) or decreasing the release of thiols (Subileau et al., 2008) depending on the study.

Interactions exist between these parameters, as the effect of the initial concentration of lipids on growth, fermentation kinetics and volatile compound profiles vary with nitrogen availability (Casalta et al., 2013). Lipid limitation is more detrimental to cell viability when the nitrogen content of the must is high (Duc et al., 2017), and a positive interaction between nitrogen and lipids in the production of isobutanol and isoamyl alcohol has been reported (Rollero et al., 2014).

As the use and study of NS yeasts is recent, little is known about their metabolism and phenotypic characteristics in fermentation or about the impact of nutrient availability on their metabolic activity. Furthermore, because of the usually poor fermentation capabilities of these other yeasts, *S. cerevisiae* is still needed to ensure the completion of fermentation. Thus, different inoculation strategies are implemented: co-inoculation, where both yeast species are inoculated at the same time in the must, or sequential inoculation, where the NS yeast is inoculated first, followed by the inoculation of *S. cerevisiae* after 1 or 2 days. In this context, nutrients are shared between the two yeast species, and thorough knowledge about the effect of substrate availability on fermentation is essential for the efficient management of the process. In fact, during sequential inoculation, the rapid consumption of nutrients present at low concentrations in the medium by NS species may prevent the implantation of *S. cerevisiae*. For example, a decrease in nitrogen availability due to *T. delbrueckii* consumption strongly decreases the growth of *S. cerevisiae* (Taillandier et al., 2014). However, these issues have been poorly addressed.

The objective of this study was to assess the effects of the initial concentrations of sugar, nitrogen and lipids on the dynamics and outcomes of sequential fermentation with *M. pulcherrima* and *S. cerevisiae*. To address this issue, a Box-Behnken experimental design (Box and Behnken, 1960) was adopted, allowing us to identify the simple and quadratic effects as well as the interaction effects of the tested variables on growth and fermentation parameters together with the production of metabolites, with a focus on aroma production and the release of varietal thiols. The same design was applied to *S. cerevisiae* in pure cultures to identify the similarities and differences between these two inoculation strategies. The results are expected to provide insights into the mechanisms underlying the influence of the tested parameters on fermentation outcomes and how to better manage these parameters during fermentation to shape the characteristics of the wine.

## Materials and Methods

### Strains and Media

*Metschnikowia pulcherrima* Flavia^®^ and *Saccharomyces cerevisiae* Lalvin QA23^®^ were provided by Lallemand SAS (Blagnac, France). The yeasts were preserved in glycerol (20% vol/vol) at –80°C. All the fermentations were carried out using a synthetic medium simulating the composition of grape juice (Bely et al., 1990) with variable contents of sugars, yeast assimilable nitrogen (YAN) and lipids, in order to analyze the effects of nutrient availability on the dynamics and outcomes of fermentation. To limit the number of experimentations, a Box–Behnken experimental design was applied (Box and Behnken, 1960), based on the use of three levels for each of the parameters to be studied (sugars, YAN, and lipids availability). To set up this experimental plan, the nutrient concentrations (Table 1) were chosen to be equally distributed within the range of concentrations found in natural grape musts reported in literature (Dharmadhikari, 1994; Deytieux et al., 2005; Palma et al., 2012) and by winemakers. A total of 15 experiments were performed (Table 2), including three at the center of the experimental domain. A base synthetic medium was prepared omitting sugar, nitrogen and lipids and divided into three batches in which different concentrations of sugars (glucose:fructose 1:1) were added: 180, 220, and 260 g/L. Each of the 3 media were subdivided into 3 parts that were further complemented with stock solutions of NH_4_Cl (100 g/L) and amino acids (Rollero et al., 2014) to achieve a final concentration of 80, 190, or 300 mgN/L. The medium was supplemented just before inoculation with phytosterols at 2, 5, or 8 mg/L. The stock solution of phytosterols was composed of 15 g/L of phytosterols (85451, Sigma Aldrich) in Tween 80 and ethanol (1:1, v/v).

**TABLE 1:** Concentrations of the studied parameters.

Parameters	Levels			Unit
	–1	0	1	
Sugar	180	220	260	g/L
Nitrogen	80	190	300	mg N/L
Lipids	2	5	8	mg/L

**TABLE 2:** Conditions tested for the Box-Behnken design.

Conditions	Sugar (g/L)	Nitrogen (mg N/L)	Lipids (mg/L)
180-80-5	180	80	5
180-190-2	180	190	2
180-190-8	180	190	8
180-300-5	180	300	5
220-80-2	220	80	2
220-80-8	220	80	8
220-190-5a	220	190	5
220-190-5b	220	190	5
220-190-5c	220	190	5
220-300-2	220	300	2
220-300-8	220	300	8
260-80-5	260	80	5
260-190-2	260	190	2
260-190-8	260	190	8
260-300-5	260	300	5

To study the production of varietal thiols, the synthetic media were supplemented with thiol precursors supplied by Nyséos (Montpellier, France) at concentrations of 105.1 μg/L of cysteinylated 3-mercaptohexan-1-ol (C3MH), 539.5 μg/L glutathionylated 3-mercaptohexan-1-ol (G3MH), 9.5 μg/L of cysteinylated 4-mercapto-4-methylpentan-2-one (C4MMP) and 10.9 μg/L glutathionylated 4-mercapto-4-methylpentan-2-one (G4MMP).

### Fermentation Conditions

Glass flasks of 330 mL were filled with 250 mL of medium and steam pasteurized for 15 min. After cooling, air was aseptically bubbled in the liquid phase under agitation for 20 min to reach an O_2_ concentration in equilibrium with the air. This step was performed to ensure the same concentration of dissolved O_2_ in all the fermenters after pasteurization. The of aeration time needed was determined by measuring the concentration of dissolved O_2_ with a non-invasive optical oxygen meter (Fibox 3 LCD trace, PreSens). The flasks were closed with fermentation locks allowing the release of CO_2_ without the entry of oxygen.

Before use, the yeasts were inoculated on YPD agar plates and incubated at 28°C for 4 days. A colony was transferred to a tube containing 5 mL of liquid YPD and incubated at 28°C under agitation for 12 h. This cell suspension was used to inoculate 20 mL of fresh YPD in an Erlenmeyer flask. The pre-culture was grown at 28°C under agitation for 24 h, and the cell concentration was measured using a Coulter counter (Model Z2, Beckman-Coulter, Margency, France). The appropriate volume for inoculation was centrifuged for 5 min at 2500 rpm, and the cells were resuspended in sterile physiological water. To comply with the practices used in winemaking, fermenters were first inoculated with *M. pulcherrima* at 1.10^7^ cells/mL. After 48 h of fermentation, *S. cerevisiae* was added to the medium at 5.10^6^ cells/mL. For pure cultures, *S. cerevisiae* was inoculated alone at 5.10^6^ cells/mL. Fermentations were performed at 22°C under agitation (280 rpm). Fermentation progress was monitored by regularly weighing the fermenters (between 2 and 5 time per day, depending on the fermentative activity): the weight loss corresponded to the amount of CO_2_ released and the rate of CO_2_ production or fermentation rate was assessed deriving the CO_2_ production over time. The fermentation was considered to be completed when the fermentation rate was lower than 0.02 g/L/h, reflecting the time at which the yeast activity stopped.

### Analytical Methods

The ammonium concentration was measured enzymatically by using a commercial kit (ENZYTECTM fluid Ammoniaque, DiaSys Diagnostic Systems Gmb). The total amino acid concentration was measured using the NOPA method (nitrogen by OPA) as described by Dukes and Butzke (1998). To determine the composition of amino acids more precisely, samples were analyzed by cation exchange chromatography with a Biochrom 30 system (Biochrom, Cambridge, United Kingdom) as described by Crepin et al. (2012).

The concentrations of glucose, fructose, ethanol, glycerol and acetate were measured by HPLC (HPLC 1290 Infinity, Agilent Technologies, Santa Clara, CA, United States) with a Phenomenex Rezex ROA column (Agilent Technologies, Santa Clara, CA, United States). The eluent solution consisted of 0.005 N H_2_SO_4_ at a flow rate of 0.6 mL/min. The acetate concentration was determined with a UV detector at 210 mm. Other compounds were detected with a refractive index detector. Data treatment was performed with the Agilent EZChrom software package.

Volatile thiol (3MH, 3MHA, and 4MMP) concentrations were measured by the company Nyséos (Montpellier, France) as described by Deroite et al. (2018). Quantification was performed via the stable isotope dilution assay. 4MMP was derivatized with EDTA, l-cysteine and *O*-methylhydroxylamine hydrochloride and then quantified using a Trace Ultra gas chromatograph (GC) equipped with a Triplus Autosampler coupled with a TSQ 8000 triple quadrupole mass spectrometer detector from Thermo Fisher Scientific (Austin, TX, United States). Data treatment was performed using Xcalibur software (Thermo Fisher Scientific, Austin, TX, United States). 3MH and 3MHA were derivatized with *N*-phenylmaleimide. Derivatized compounds were injected onto a ProtID HPLC-polymeric chip (ZORBAX 300SB-C18, 40 nL, 5 μm and 75 μm * 43 μm, 5 μm). Compounds were eluted with Agilent Series 1260 Infinity nano-LC (Agilent, Santa Clara, CA, United States) connected to an Agilent 6460 triple quadrupole mass spectrometer (Agilent Technologies, Waldbronn, Germany). Data analysis was performed with Agilent Mass Hunter ChemStation software.

Fermentative aromas were measured by GC-MS following the method described by Rollero et al. (2014). Ten microliters of a solution of the deuterated standard at 100 ng/L was added to 5 mL of sample. Volatile compounds were extracted using 1 mL of dichloromethane. After shaking for 20 min and 5 min of centrifugation at 5 000 rpm at 4°C, the organic phase was retrieved. This step was repeated for the aqueous phase. The total organic phase was dried with Na_2_SO_4_ and evaporated under nitrogen flux to obtain a final volume of 0.5 mL.

Samples were analyzed in a Hewlett Packard 6890 (Agilent Technologies, Santa Clara, CA, United States) gas chromatograp equipped with a CTC Combi PAL AOC-5000 (Shimadzu, Colombia, Etats-Unis) autosampler and an HP 5973 (Agilent Technologies, Santa Clara, CA, United States) mass spectrometry detector. The gas chromatograph was equipped with a DB-WAX (30 m ^∗^ 0.250 mm ^∗^ 0.25 μm, Agilent J&W) column. The carrier gas was helium applied at a flow rate of 1.0 mL/min and a linear velocity of 36 cm/s in constant flow mode. The initial oven temperature was 40°C for 3 min, which was then increased to 160°C at a rate of 4°C/min and finally to 220°C at 15°C/min, then kept at 220°C for 10 min. The mass spectrometry detection parameters were previously described by Rollero et al. (2014). Response factors were calculated from calibration curves obtained with standard solutions prepared in hydroalcoholic solutions containing volatile molecules with various concentrations (Table 3), processed at the same time and via the same method as the samples.

**TABLE 3:** Concentration ranges (in mg/L) of volatile compounds measured by GC-MS.

	Concentration range (mg/L)	
	Minimum	Maximum
Propanol	0.50	20.12
Isobutanol	0.59	29.88
Isoamyl alcohol	0.78	39.30
Phenylethanol	0.66	33.28
Isobutyl acetate	0.05	2.49
Isoamyl acetate	0.05	2.81
Phenylethyl acetate	0.06	3.43
Ethyl isobutyrate	0.05	2.65
Ethyl hexanoate	0.07	3.72
Ethyl octanoate	0.05	2.97
Ethyl decanoate	0.05	2.68
Isobutyric Acid	0.04	2.23
Hexanoic Acid	0.42	17.08
Octanoic Acid	0.41	20.80
Decanoic Acid	0.05	2.14

All the analytic determinations were performed in duplicate.

### Measure of β-Lyase Activity

β-lyase activity was evaluated in a colorimetric reaction described by Roncoroni et al. (2011). The determination was based on the quantification of the amount of ethanethiol produced from the degradation of the specific substrate S-ethyl-cysteine by β-lyase enzymes. The reaction was performed by mixing 250 μL of S-ethyl-cysteine, 100 μL of reaction buffer, 250 μL of DTNB and 250 μL of culture supernatant. The reaction buffer was prepared with EDTA at 0.1 mmol/L and PLP (a coenzyme) at 0.1 mmol/L in McIlvaine buffer (pH 3.4). To avoid interference with other sulfur compounds potentially produced during fermentation, a blank was set up by replacing the S-ethyl-cysteine with the same volume of water. After 2 h of incubation at 28°C under agitation, the mixture was centrifuged before reading the absorbance at 400 nm. The increase in absorbance was proportional to the amount of ethanethiol produced by the enzymatic reaction.

Total activity was calculated as the area under the curve of the activity by the fermentation time. This area was estimated with the trapezoidal rule. All the β-lyase activity determinations were performed in triplicate.

### Data Treatment

The model associated with the Box-Behnken design was applied to investigate the impact of the three independent variable on 6 parameters describing the fermentation process and on the production of 22 metabolites.

The main parameters describing the fermentation kinetics were calculated from the measured CO_2_ production over time. The fermentation duration corresponded to the time needed to reach the theoretical final CO_2_ concentration depending on the initial sugar content of the medium. The lag phase duration was calculated as the intersection between the x-axis and the tangent at the inflection point of the CO_2_ production curve. The time derivative of the CO_2_ production was calculated to obtain the fermentation rate. In pure culture, the maximal value of the fermentation rate was referred to as R_max_, and the time required to reach this rate was referred to as T_Rmax_. Under sequential inoculations, the maximal fermentation rate obtained before the inoculation of *S. cerevisiae* was referred to as R_max,MP_ and the time required to reach it as T_Rmax,MP_. Conversely, the maximal fermentation rate obtained after the inoculation of *S. cerevisiae* was referred to as R_max,SC_, and the time required to reach it was T_Rmax,SC_.

Statistical analysis and response surface representation were performed using R (version 3.4.3) and rsm library (Lenth, 2009).

The effect of the parameters on the experimental results was modeled with a polynomial response surface:

$$Y=\beta_{0}+\beta_{1}\,x_{1}+\beta_{2}\,x_{2}+\beta_{3}\,x_{3}+\beta_{1,2}\,x_{1}\,x_{2}+\beta_{1,3}\,x_{1}\,x_{3}+\beta_{2,3}\,x_{2}\,x_{3}+\beta_{1,1}\,x_{1}^{2}+\beta_{2,2}\,x_{2}^{2}+\beta_{3,3}\,x_{3}^{2}+\varepsilon$$

where:

Y: observed result

x_1_, x_2_, x_3_: coded values of sugar, nitrogen and lipids concentrations

β_0_: intercept term

β_i_: linear coefficient

β_i,j_: interaction coefficient

β_i,i_: quadratic coefficient

ε: independent error term

The reliability of the model was evaluated by the lack of fit test, the Fisher test and the adjusted coefficient of determination, adjR^2^. According to these indicators, the model was simplified by removing the interaction and/or the quadratic term if necessary.

Principal component analysis (PCA) was carried out with the FactoMineR package (Le et al., 2008).

## Results

To address the issue of the effect of nutrient availability on fermentation progress under *M. pulcherrima*/*S. cerevisiae* sequential inoculation, the growth and fermentation performance as well as the production profiles of carbon central metabolites (CCM) and volatile compounds were compared between *S. cerevisiae* pure culture and sequential fermentation using variable concentrations of lipids, sugars and nitrogen in both cases. The production of thiols and β-lyase activity were assessed by supplementing the synthetic must with thiol precursors. To optimize the number of experiments necessary to test three factors at three levels, a Box-Behnken experimental design was used consisting of fifteen fermentations under thirteen different conditions, as summarized in Table 2. By fitting the results with the model equation, we can determine the effect of each factor on the studied parameters as well as the interactions between the factors.

Regardless of the inoculation strategy and nutrient availability, all the fermentations reached dryness, with a residual sugar concentration lower than 2 g/L at the end of culture.

### Fermentation Kinetics

**FIGURE 1:**
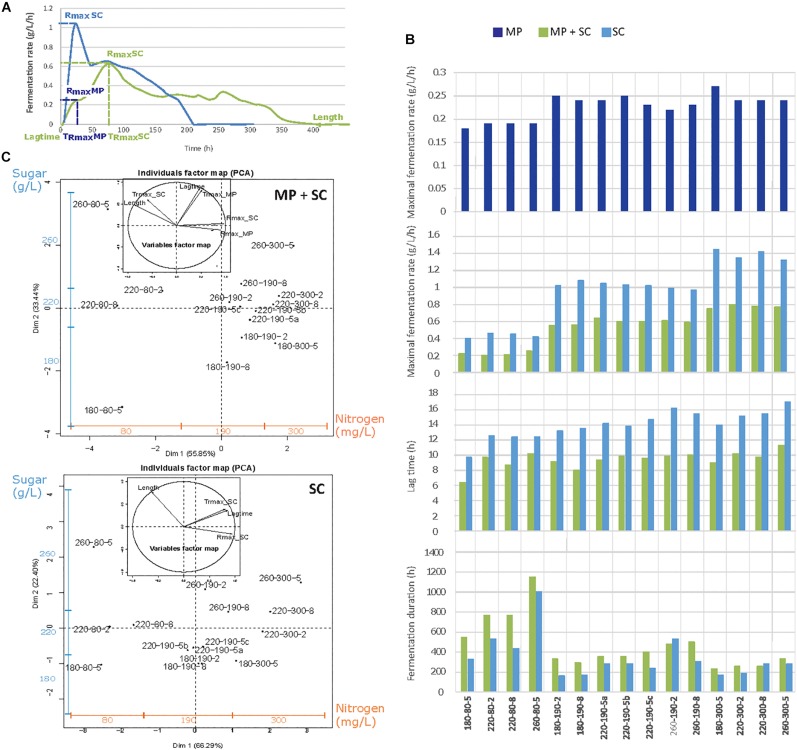
**(A)** Example of the fermentation kinetics of *M. pulcherrima* and *S. cerevisiae* under sequential inoculation (MP + SC, green line) and *S. cerevisiae* in pure culture (SC, blue line). **(B)** Parameters describing the fermentation kinetics depending on the medium composition and inoculation strategy: Rmax_MP, maximal fermentation rate for *M. pulcherrima* reached before the inoculation of *S. cerevisiae*; Rmax_SC, maximal fermentation rate for *S. cerevisiae*; lag phase duration and fermentation duration. MP, dark blue bars: pure culture of *M. pulcherrima*. MP + SC, green bars: sequential fermentation. SC, blue bars: pure culture of *S. cerevisiae*. Conditions are labeled as sugar-nitrogen-lipids. **(C)** Principal component analysis (PCA) of fermentative parameters under the sequential inoculation of *M. pulcherrima* and *S. cerevisiae* and in pure cultures of *S. cerevisiae*. Individuals are identified as S-N-L, where S, sugar concentration; N, nitrogen concentration; and L, lipid concentration. The blue and orange additional axes indicate the repartition of individuals according to the concentrations of sugar (180–220–260 g/L) and nitrogen (80–190–300 mg/L). The inserts correspond to the correlation circles representing the explanatory variables. Trmax_MP and Trmax_SC, time to reach Rmax before and after the inoculation of *S. cerevisiae*.

Substantial differences in the fermentation kinetics were observed between *S. cerevisiae* pure culture and sequential fermentation with *M. pulcherrima* and *S. cerevisiae* (Figure 1A). As widely reported (Bely et al., 1990; Mendes-Ferreira et al., 2004; Beltran et al., 2005), the rate of *S. cerevisiae* fermentation was maximal after approximately 24 h, after which it decreased continuously throughout the process. In contrast, fermentative activity evolved in two steps during sequential fermentation, showing a first maximum during the first 48 h, when only *M. pulcherrima* supported fermentation, followed by a second maximum after inoculation with *S. cerevisiae* and corresponding to a higher fermentation rate.

To provide an overview of the effect of nutrients on fermentation progress, the main fermentation traits (maximal fermentation rate before and after *S. cerevisiae* inoculation, R_max_ MP and R_max_ SC, respectively; the time to reach this maximum, T_Rmax_; the lag phase duration; and the fermentation duration) were measured under sequential inoculation and in pure culture (Figure 1B) and represented using principal component analysis (PCA) (Figure 1C). Similar responses were obtained for the two inoculation protocols, for which the two first axes of the PCA representation explained 89% of the total variance. Individuals were separated depending on both the nitrogen concentration along the horizontal axis and the sugar concentration along the vertical axis. Overall, increased nitrogen availability led to a higher R_max_ before and after *S. cerevisiae* inoculation resulting in a reduced fermentation duration (Length). Otherwise, an increase in the sugar concentration led to an increase in the lag phase (Lag time) and fermentation durations, as well as an increase in the time to reach the maximal fermentation rate, both before and after *S. cerevisiae* inoculation (T_rmax_MP_; T_rmax_SC_).

A more thorough analysis revealed the consequences of early inoculation with *M. pulcherrima* for fermentation performance. First, the maximal fermentation rate during sequential fermentations, varying from 0.2 to 0.8 g/L/h depending on nitrogen availability, was always lower than that measured in *S. cerevisiae* pure cultures (between 0.4 and 1.4 g/L/h) (Figure 1B). It is noteworthy that the increase in R_max_ with the nitrogen concentration was less pronounced during sequential fermentation (+0.6 g/L/h, Figure 1B) compared with pure culture modality (+1.0 g/L/h, Figure 1B) and that nitrogen availability had no impact on the fermentation rate of *M. pulcherrima* (Supplementary Data S1A).

During *S. cerevisiae* fermentation, the lag phase (Figure 1B) was between 10 and 16 h and shorter when nitrogen was limiting (80 mg N/L). This effect of nitrogen availability was not found under *M. pulcherrima*/*S. cerevisiae* sequential fermentation, for which the lag phase varied from 6 to 11 h. Finally, nutrient availability had the same effect on the duration of sequential fermentation and pure cultures, with a substantial increase in the duration when nitrogen was limiting (80 mg N/L). An interaction between nitrogen and the initial sugar concentration was observed, with a more pronounced effect of the sugar concentration on the fermentation duration under a low nitrogen concentration compared with the other conditions (190 and 300 mg N/L, Figure 1B).

### Nitrogen Consumption and Biomass Production

**FIGURE 2:**
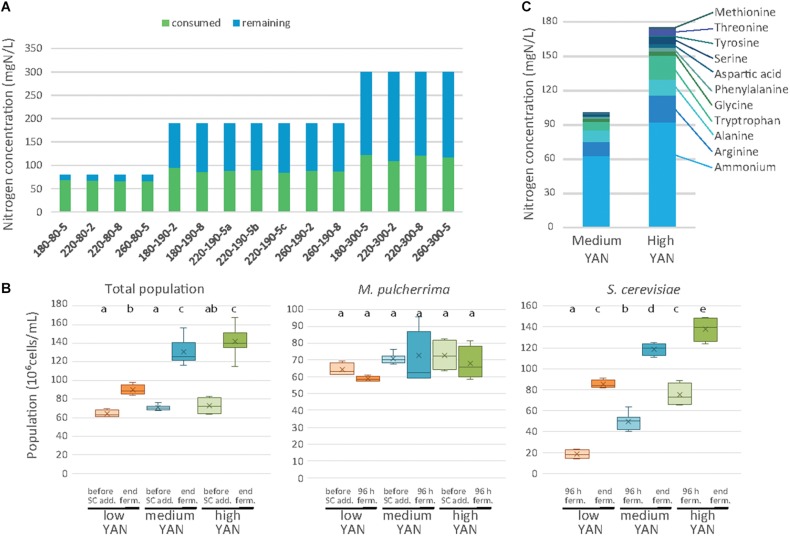
Nitrogen consumption and cell populations during sequential fermentations. **(A)** Concentrations of consumed (blue bars) and residual (green bars) assimilable nitrogen after 48 h of fermentation with *M. pulcherrima*, depending on the conditions. Conditions are labeled as sugar-nitrogen-lipids. **(B)** Total cell population, *M. pulcherrima* population and *S. cerevisiae* population depending on the nitrogen availability (low: orange, medium: blue, high: green) at different stages of fermentation. **(C)** Composition of the nitrogen resource after 48 h of *M. pulcherrima f*ermentation in presence of medium or high YAN concentration.

The growth of the two species was investigated in association with the nitrogen consumption profile during pure and sequential fermentations (Figure 2). The residual nitrogen concentration in the medium was first measured after 48 h of fermentation with *M. pulcherrima* before the inoculation of *S. cerevisiae* (Figure 2A). The consumption of nitrogen by *M. pulcherrima* was strongly dependent on the initial YAN concentration and accounted for 84, 47 and 39% of the nitrogen provided during fermentation with 80, 190, or 300 mg N/L, respectively. However, the amount of consumed nitrogen (between 65 and 122 mg N/L) did not significantly affect the *M. pulcherrima* population after 48 h of fermentation, which displayed similar levels regardless of nutrient availability (Figure 2B). Finally, it is noteworthy that no significant increase in the *M. pulcherrima* population was found after *S. cerevisiae* addition (Figures 2A,B), indicating that *M. pulcherrima* did not grow after 48 h in sequential fermentation.

While almost entirely consumed by *M. pulcherrima* when nitrogen was limiting, the nitrogen sources can be differentiated on the basis of their level of consumption (Supplementary Data S1) depending on the nitrogen concentration (190 or 300 mg N/L). Branched amino acids, glutamine, glutamate and lysine were exhausted 48 h after *M. pulcherrima* addition, regardless of the initial YAN concentration. Other amino acids such as arginine, serine and threonine were largely consumed in the presence of 190 mg N/L, but their assimilation remained incomplete (55%) when 300 mg N/L was supplied in the medium. Finally, only a fraction of the provided arginine, ammonium, alanine and aromatic amino acids was consumed when nitrogen was present in excess in the medium. As a result, the composition of the nitrogen resources when *S. cerevisiae* was added differed according to the initial YAN concentration, even though ammonium, arginine, alanine and tryptophan were the most abundant compounds in all the conditions (Figure 2C and Supplementary Data S1B, S2).

The concentration of remaining nitrogen had a substantial effect on the implantation of *S. cerevisiae* (Figures 2B,C and Supplementary Data S2). The population of *S. cerevisiae* 48 h after inoculation was small under conditions with 80 mgN/L of initial nitrogen (18 10^6^ cells/mL on average), consistent with the low amount of available YAN (less than 13 mg N/L), but the population reached 50 10^6^ cells/mL and 75 10^6^ cells/mL in the presence of 190 and 300 mg N/L, respectively. At the end of fermentation, the *S. cerevisiae* population varied between 82 and 150 10^6^ cells/mL according to the initial nitrogen availability, while no viable *M. pulcherrima* cells were detected (Figure 2B). It is noteworthy that this response to changes in nitrogen availability in terms of the final population was similar to that observed in *S. cerevisiae* pure cultures, in which the final population increased from 72 to 185 10^6^ cells/mL with an increase in the initial nitrogen content from 80 to 300 mg/L (Supplementary Data S3).

### Production of Ethanol, Acetate, and Glycerol

**FIGURE 3:**
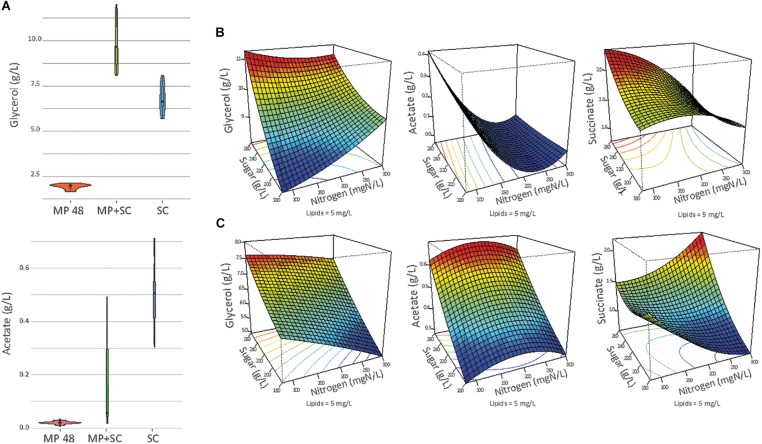
**(A)** Production of glycerol and acetate in g/L at the end of fermentation (or at 48 h of fermentation if specified) in pure culture of *S. cerevisiae* (SC), under sequential inoculation at 48 h (MP 48 h) or at the end of the fermentation (MP + SC). At 48 h of fermentation, only *M. pulcherrima* is present in the medium. **(B)** Response surfaces of glycerol, acetate and succinate for fermentation under sequential inoculation **(A)** or in pure culture of *S. cerevisiae* **(B)** in relation to the variation of sugar, lipids and nitrogen. For all graphs, the lipid concentration is fixed at 5 mg/L.

The production of ethanol, acetate and glycerol was measured after 48 h of fermentation with *M. pulcherrima* and at the end of fermentation under sequential inoculation and in pure culture of *S. cerevisiae* (Figure 3A).

First, the production of ethanol at the end of fermentation was lower under sequential inoculation than in pure culture of *S. cerevisiae* (Table 4). The difference was 8-9 g/L on average, regardless of the composition of the medium, while the sugar consumption was similar for the two inoculation modalities. This observation is consistent with the differences in the ethanol production yield between the studied species. We observed a lower ethanol production yield for *M. pulcherrima* (0.424 g_EtOH_/g_sugar_, calculated 48 h before the inoculation of *S. cerevisiae*) compared to *S. cerevisiae* (0.487 g_EtOH_/g_sugar_ during pure culture).

**TABLE 4:** Concentrations of ethanol in g/L and in %vol at the end of fermentation under sequential inoculation (MP + SC) or in pure culture (SC) depending on the initial sugar concentration in the medium.

	Mean ethanol					
	concentration (g/L)			Mean abv (%vol)		
Initial sugar concentration (g/L)			Difference%			
	MP + SC	SC		MP + SC	SC	Difference
180	80.97	89.57	10%	10.3	11.4	1.1
220	102.58	110.83	7%	13.0	14.0	1.0
260	123.28	131.23	6%	15.6	16.6	1.0

The lower formation of ethanol during sequential inoculation was combined with increased formation of glycerol and succinate by up to +4.7 g/L and +1.1 g/L, respectively (Supplementary Data S4). Therefore, the production of glycerol in the presence of *M. pulcherrima* was between 8.1 and 12.0 g/L and was higher than the formation of glycerol during *S. cerevisiae* pure fermentation (5.7 and 8.1 g/L), irrespective of the initial must composition (Supplementary Data S4). This observation has to be considered first in the context of the high capacity of *M. pulcherrima* to produce glycerol during the first part of the fermentation (between 1.65 and 2.14 g/L of glycerol produced after 48 h). Furthermore, after *S. cerevisiae* inoculation, the glycerol production measured during sequential inoculation (0.046 g_glycerol_/g_sugar_), to be exclusively attributed to *S. cerevisiae* as the only growing yeast, was higher than in *S. cerevisiae* pure cultures (0.031 g_glycerol_/g_sugar_).

In contrast, acetate production was considerably decreased when *M. pulcherrima* was inoculated first. The final concentrations of acetate were between 0.03 and 0.49 g/L, with a mean value of 0.16 g/L, under sequential inoculation and between 0.30 and 0.71 g/L, with a mean value of 0.51 g/L, in pure culture. This was likely related to the low capacity of *M. pulcherrima* to produce acetate (0.01 g/L to 0.03 g/L produced in 48 h). Interestingly, during fermentation with 190 or 300 mg N/L, the production of acetate remained limited after *S. cerevisiae* inoculation (yield < 0.2 mg_acetate_/gs_ugar_) and did not exceed 0.06 g/L at the end of sequential fermentation. In contrast, when nitrogen was limiting, the yield of acetate production after *S. cerevisiae* inoculation was higher, ranging from 1.7 to 2.2 mg_acetate_/g_sugar_, resulting in a final acetate concentration of 0.4 g/L.

This observation indicated differential production of acetate during sequential fermentation depending on the initial YAN concentrations. Then, to obtain an overview of the effect of nutrient availability on the production of glycerol, acetate and succinate, the dataset was analyzed with a quadratic model fitting the response surfaces from the Box-Behnken design (Figure 3B and Table 5). For *S. cerevisiae* in pure culture, a positive effect of sugar on the production of glycerol, succinate and acetate was observed, combined with simple or quadratic negative effects of nitrogen on glycerol and acetate, respectively. Last, a positive interaction effect between sugar and nitrogen was observed on the production of succinate. The implementation of sequential inoculation with *M. pulcherrima* resulted in important changes in the impact of nutrient availability on the formation of glycerol, acetate and succinate. Most notably, the negative effect of nitrogen on glycerol production observed in *S. cerevisiae* in pure culture was reversed by the presence of *M. pulcherrima*, as a positive effect of nitrogen availability was found under sequential fermentation (Figure 3B). Moreover, an interaction between nitrogen and sugar occurred, as the effect of nitrogen was positive when the concentration of sugar was low but was slightly negative when the concentration of sugar was high. Otherwise, the strong effect of nitrogen on the production of acetate during sequential fermentation was confirmed by the evidence of a negative simple effect of nutrient combined with a slight positive quadratic effect. This pattern contrasted with that observed in *S. cerevisiae* pure culture, in which sugar availability exerted the main effect.

**TABLE 5:** Effects of must parameters on the production of glycerol and acetate by *M. pulcherrima* in pure culture after 48 h of fermentation (MP), *M. pulcherrima* and *S. cerevisiae* under sequential inoculation (MP + SC) and *S. cerevisiae* in pure culture (SC).

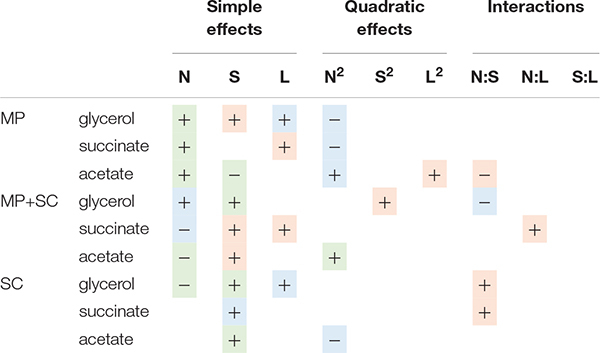

*+, positive effect; −, negative effect. Green, *p* < 0.001; blue, *p* < 0.01; and orange, *p* < 0.05. *N*, nitrogen; S, sugar; L, lipids.*

### Fermentative Aromas

**FIGURE 4:**
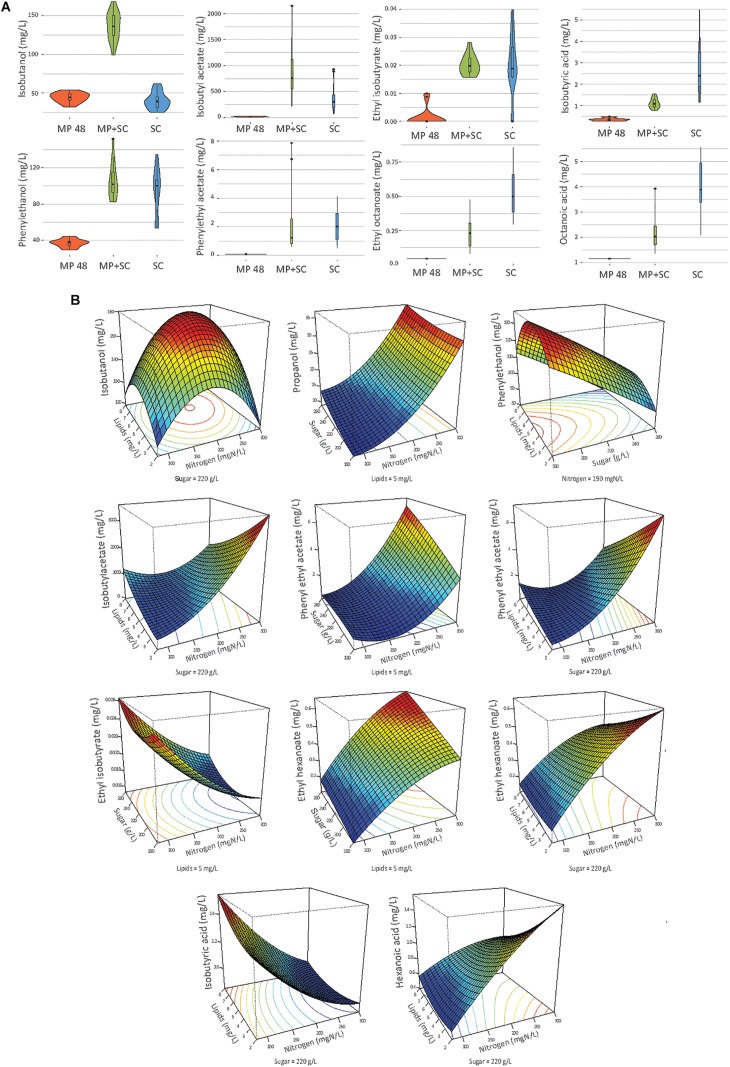
**(A)** Combined violin and box plot representation of some aroma compounds produced by *M. pulcherrima* in pure culture after 48 h of fermentation (MP), *M. pulcherrima* and *S. cerevisiae* under sequential inoculation after 90% of fermentation progress (MP + SC) and *S. cerevisiae* in pure culture after 90% of fermentation progress (SC). **(B)** Response surfaces of fermentative aromas in relation to the variation of sugar, lipids and nitrogen under sequential inoculation. For all graphs, one parameter is fixed.

The variability in the production of fermentative aromas depending on both the inoculation procedure and nutrient availability was then assessed by comparing the production of 30 volatile compounds after 48 h of *M. pulcherrima* fermentation and at the end of sequential fermentation or *S. cerevisiae* pure culture (Figure 4A). The complete dataset (Supplementary Data S5, S6) was analyzed with the model associated with the Box-Behnken design (Table 6) and represented with response surfaces (Figure 4B and Supplementary Data S7).

**TABLE 6:** Effects of must parameters on the production of the main fermentative aromas by *M. pulcherrima* and *S. cerevisiae* under sequential inoculation (MP+SC) and *S. cerevisiae* in pure culture (SC).

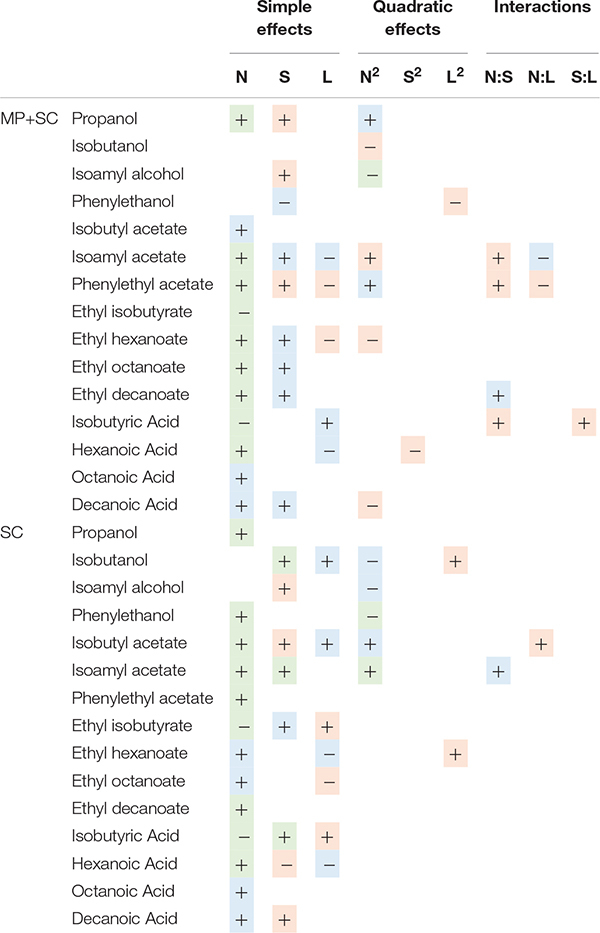

*+, positive effect; −, negative effect. Green, *p* < 0.001; blue, *p* < 0.01; and orange, *p* < 0.05. *N*, nitrogen; S, sugar; L, lipids.*

The production of fatty acids and their corresponding ethyl esters was lower under sequential inoculation than in *S. cerevisiae* pure culture (Figure 4A). This difference was increased for fatty acids with the longest chains, with 2-fold and 4.7-fold decreases in the production of hexanoic and decanoic acids, respectively. Overall, the production of these two families of volatile molecules showed the same response to environmental parameter changes irrespective of the inoculation protocol (Table 6). The formation of medium-chain fatty acids and their ethyl ester derivatives was positively affected by the sugar concentration but was mainly affected by nitrogen availability, while lipid content in the medium had a very limited effect (Figure 4B). A negative effect of lipids was observed only for the production of the compounds with the shortest carbon chains, specifically for the production of ethyl hexanoate and hexanoic acid under sequential inoculation and the production of ethyl hexanoate, hexanoic acid and ethyl octanoate in pure culture of *S. cerevisiae* (Figure 4B, Table 6, and Supplementary Data S7).

The effects of sugar, nitrogen and lipids on the compounds produced through the Ehrlich pathway depend on the metabolic route involved in the formation of their precursor.

The production of propanol and its derivatives (propyl acetate, propionic acid and ethyl propionate) was similar regardless of the inoculation strategy (Figure 4A). Only the production of propionic acid was lower under sequential inoculation with *M. pulcherrima*. These compounds showed similar responses to changes in nutrient availability, characterized mainly by higher production with higher nitrogen availability (Table 6).

The final concentration of compounds from the isobutanol family was highly impacted by the presence of *M. pulcherrima* (Figure 4A). Indeed, the production of isobutanol in pure culture of *S. cerevisiae* was between 25 and 62 mg/L, while under sequential inoculation, it ranged between 99 and 167 mg/L. The production of isobutyl acetate was also 3 times higher in cultures with *M. pulcherrima* than in pure cultures of *S. cerevisiae*. In contrast, the production of isobutyric acid was 2.5 times lower under sequential inoculation. The three tested parameters affected the production of these compounds by *S. cerevisiae* under pure culture conditions (Supplementary Data S7). However, almost only nitrogen had an effect on the production of these compounds under sequential inoculation (Figure 4B). Nitrogen had a quadratic effect on the production of isobutanol, where maximal production was observed in the presence of 200 mgN/L of assimilable nitrogen. The production of isobutyl acetate increased with nitrogen availability. In contrast, the production of isobutyric acid decreased with increasing concentrations of nitrogen. Only the production of isobutyric acid was positively impacted by the concentration of lipids.

The effect of sequential inoculation with *M. pulcherrima* on the production of compounds derived from isoamyl alcohol was less pronounced than it was for isobutanol. The production of isoamyl alcohol was increased by an average of 15%, and the production of isoamyl acetate was increased by 44% under sequential inoculation. Consequently, the effect of *M. pulcherrima* inoculation on the way in which the nutrient concentration impacted the production of these compounds was weak (Figure 4B and Table 6). Nitrogen had a quadratic effect on the production of isoamyl alcohol and a positive effect on the production of isoamyl acetate, which interacted with a negative effect of lipids. Finally, we observed a positive effect of sugar on the production of all these compounds.

The production of phenyl ethanol under sequential inoculation was higher than in pure culture of *S. cerevisiae* in the conditions with low levels of nitrogen and similar to that when nitrogen availability was higher (Figure 4 and Supplementary Data S7). The production of this compound in pure culture is impacted by the nitrogen concentration in a quadratic manner, but the effect of nitrogen disappears under fermentation with *M. pulcherrima*. In contrast, a positive effect of nitrogen on the production of phenyl ethyl acetate was present under both inoculation conditions. In addition, phenyl ethyl acetate production was subject to a positive effect of sugar and a positive interaction between sugar and nitrogen as well as a negative effect of lipids and a negative interaction between lipids and nitrogen.

### Varietal Thiols

**FIGURE 5:**
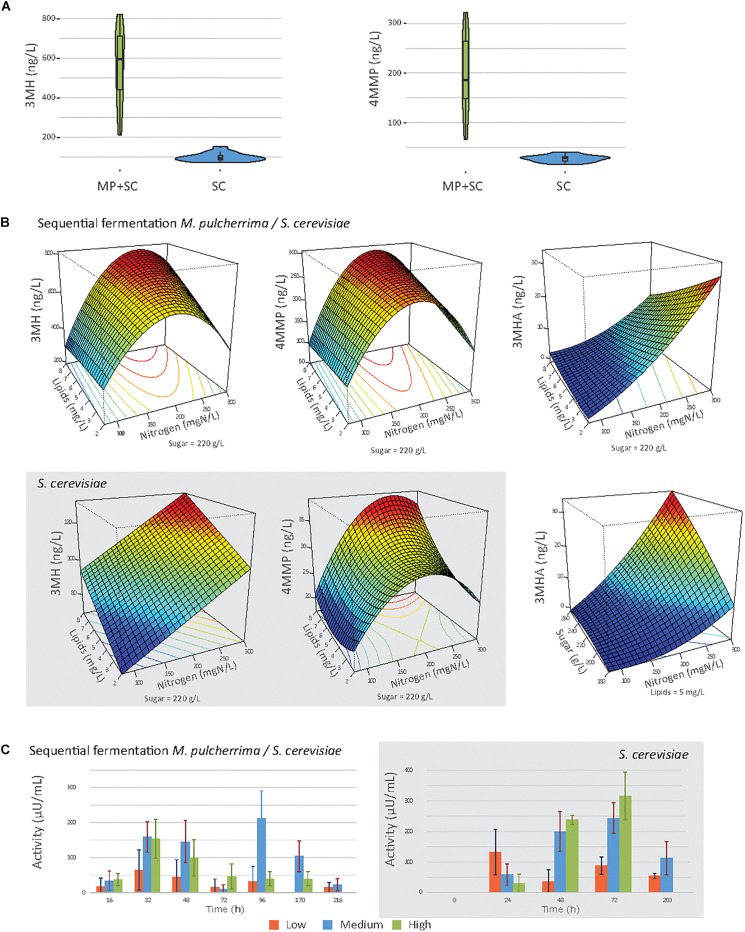
**(A)** Violin plot of the production of 3MH and 4MMP under sequential inoculation (MP + SC) or in pure culture of *S. cerevisiae* (SC), at 90% of fermentation progress. **(B)** Response surfaces of thiol production under sequential inoculation or in pure culture of *S. cerevisiae* (gray box). **(C)** Mean β-lyase activity depending on the initial nitrogen concentration during fermentation under sequential inoculation and in pure culture of *S. cerevisiae* (gray box). 80 mgN/L: orange bars, 190 mN/L: blue bars, and 300 mgN/L: green bars.

Finally, we investigated the incidence of the inoculation procedure and nutrient availability on thiol liberation during wine fermentation (Figure 5 and Table 7).

**TABLE 7:** Effects of must parameters on the release of thiols by *M. pulcherrima* and *S. cerevisiae* under sequential inoculation (MP+SC) and *S. cerevisiae* in pure culture (SC).

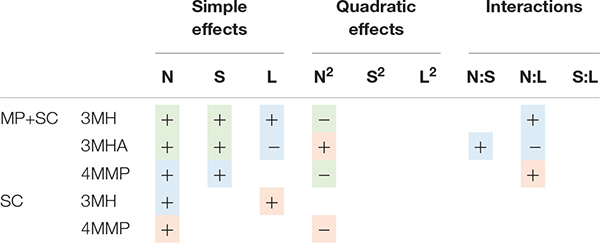

*+, positive effect; -, negative effect. Green, *p* < 0.001; blue, *p* < 0.01; and orange: *p* < 0.05. *N*, nitrogen; S, sugar; L, lipids.*

Regardless of the fermentation conditions, the sequential inoculation of *M. pulcherrima* and *S. cerevisiae* led to a marked increase in thiol release compared to that in *S. cerevisiae* pure culture (Figure 5A). Indeed, in pure culture of *S. cerevisiae*, the production of 3MH was between 72 and 152 ng/L, and the production of 4MMP was between 16 and 41 ng/L. No 3MHA was produced. Under sequential inoculation, the production of 3MH was between 273.7 and 821.3 ng/L, and the production of 4MMP was between 66.0 and 326.2 ng/L. 3MHA was produced in some conditions, at levels up to 32.3 ng/L. It is also noteworthy that after 48 h of fermentation with *M. pulcherrima* in pure culture, no thiol release was measured.

High variability was observed in the production of thiols according to nutrient availability, especially under sequential inoculation with *M. pulcherrima* (Figure 5B). The production of thiols was mainly impacted by the initial amount of nitrogen (Table 7). We observed a quadratic impact of nitrogen on the production of 3MH and 4MMP. The production of these compounds increased up to an initial nitrogen concentration of 200 mg N/L and decreased with higher concentrations. The impact of nitrogen on the production of 3MHA was positive irrespective of the concentration of nitrogen. Under sequential inoculation, the initial concentration of sugar also had a positive impact on the production of thiols. The production of 3MH was positively impacted by the concentration of lipids in the medium. We also noticed an interaction between nitrogen and lipids, which was positive for the production of 3MH and 4MMP and negative for the production of 3MHA.

β-lyase activity, produced by yeasts and responsible for the release of thiols from their non-aromatic precursors, was followed during fermentation (Figure 5C). Under sequential inoculation, β-lyase activity reached two maxima. The first one occurred between 24 and 48 h depending on the conditions. The activity then decreased before increasing again and reaching a second maximum at approximately 96 h of fermentation. In contrast, β-lyase activity in pure culture of *S. cerevisiae* showed only one maximum. This maximum was reached at 24 h of fermentation under conditions with 90 mgN/L of initial nitrogen and between 48 and 72 h of fermentation for the other conditions. When the activities were compared between sequential inoculation and the pure culture of *S. cerevisiae*, the same magnitude of activity was observed. Nitrogen availability seemed to be the parameter with the main impact on β-lyase activity.

## Discussion

Currently, the use of NS yeasts in mixed fermentation with *S. cerevisiae* has become a ubiquitous practice in winemaking, with the aim of improving the complexity of wines. Thus, it has been reported that early inoculation of *M. pulcherrima* results in changes in the characteristics of wines (Rodríguez et al., 2010; González-Royo et al., 2015; Varela et al., 2016). However, this procedure may also affect fermentation performance and the effect of environmental parameters on the both process and profile of carbon metabolite and volatile compound production may differ between sequential inoculation and pure culture.

### Nitrogen Consumption by *M. pulcherrima* Alters the Fermentation Kinetics Under Sequential Inoculation

We first found that sequential fermentation with *M. pulcherrima* associated with *S. cerevisiae* took longer than fermentation in *S. cerevisiae* pure culture, lasting up to 250 additional hours, regardless of the lipids, nitrogen and sugar concentrations in the medium. The poor fermentation capabilities of *M. pulcherrima*, for which the fermentation rate did not exceed 0.27 g/L/h, combined with the delay of *S. cerevisiae* inoculation, were the first elements to be taken into account to explain this observation. In addition, the consumption of nutrients, particularly nitrogen, by *M. pulcherrima* during the first 48 h of the process may also play a part in lengthening the duration of sequential fermentation. Indeed, early inoculation with a NS yeast strain often results in decreased *S. cerevisiae* fermentation performance due to nitrogen limitation (Medina et al., 2012; Taillandier et al., 2014; Rollero et al., 2018). During *M. pulcherrima*/*S. cerevisiae* sequential fermentation, this is of particular relevance in the presence of low initial YAN levels. In fact, when nitrogen was limiting (80 mg/L), the substantial consumption of nitrogen sources during the *M. pulcherrima* pure culture stage, up to 80% of the provided YAN, prevented the further implantation of *S. cerevisiae*, leading to a slow-down of fermentation. In the case of moderate and high initial YAN contents, the residual nitrogen level was higher at the time of *S. cerevisiae* addition, between 95 and 190 mg N/L, but some specific nitrogen sources, such as branched amino acids, glutamate and glutamine, were exhausted, in accordance with the sequence of the consumption of nitrogen sources by *M. pulcherrima* previously reported by Su et al. (2020). As a consequence, the nitrogen resources available to promote further *S. cerevisiae* implantation were mainly composed of proline, tryptophan, glycine and alanine, which support the growth and fermentation of the yeast poorly (Godard et al., 2007; Fairbairn et al., 2017). Overall, early inoculation with *M. pulcherrima* resulted in a 1.7- to 2-fold decrease in the maximal fermentation rate of *S. cerevisiae*. Overall, these results revealed that appropriate management of yeast nutrition during *M. pulcherrima/S. cerevisiae* sequential fermentation, such as the addition of nitrogen at the time of the inoculation of *S. cerevisiae*, can be beneficial to achieve the completion of fermentation, as previously recommended for co-fermentation with other non-conventional species (Medina et al., 2012; Rollero et al., 2018). Furthermore, other parameters have to be taken into account to explain the incidence of an early inoculation of *M. pulcherrima* on *S. cerevisiae* growth, including a limitation in vitamin that are rapidly consumed during fermentation (Medina et al., 2012; Rollero et al., 2018) or the production of pulcherriminic acid, an iron chelating agent (Sipiczki, 2006; Oro et al., 2014).

As a general rule, the lag phase of sequential fermentation during which *M. pulcherrima* was the only yeast in the medium was shorter than that of *S. cerevisiae* pure culture. This result was consistent with previous findings indicating a shorter lag phase for *M. pulcherrima* (Su et al., 2020). The changes in the lag phase duration in response to changes in nutrient availability were similar for both strains. The increase in the lag phase associated with the sugar concentration may be explained by higher osmotic stress in the presence of high sugar concentrations, as delayed growth and fermentation have been observed in response to an increase in osmotic stress (Slaninová et al., 2000; Hohmann, 2002; Mager and Siderius, 2002).

### Interactions Between *M. pulcherrima* and *S. cerevisiae* Change the Distribution of Carbon Fluxes

One of the main reasons for the renewed interest in NS yeasts is the potential of some species used in combination with *S. cerevisiae* during wine fermentation to reduce the ethanol content of wine (Contreras et al., 2014; Quirós et al., 2014; Canonico et al., 2016). The effectiveness and feasibility of these approaches may depend on the species, strains and fermentation conditions involved. Under varying initial concentrations of lipids, sugar and nitrogen, we observed that sequential fermentation involving *M. pulcherrima* and *S. cerevisiae* strains resulted in a reduction in the ethanol level by 8 g/L on average compared to *S. cerevisiae* pure culture (Table 5). This is in agreement with results in the literature indicating decreases between 7.1 and 13.2 g/L according to the species (Contreras et al., 2015; Ciani et al., 2016). Interestingly, no significant effect of nutrient availability on the reduction in ethanol content during *M. pulcherrima*/*S. cerevisiae* sequential fermentation was evidenced.

A substantial decrease in ethanol production inevitably implies carbon rerouting toward the formation of other central metabolites to maintain both mass and redox balances during fermentation. Thus, higher production of glycerol, with an average increase of 50%, was found for *M. pulcherrima* under sequential inoculation compared with *S. cerevisiae* in pure culture, as previously reported (Comitini et al., 2011; Hranilovic et al., 2018). This observation can be explained first by the high capacity of *M. pulcherrima* to produce glycerol during the first part of the fermentation. Furthermore, it may be related in part to the positive interaction between the two yeast species, promoting glycerol production by *S. cerevisiae*.

Sugar availability, controlling the glycolytic flux (Klein et al., 1998), modulated glycerol production during sequential fermentation and *S. cerevisiae* pure culture similarly. On the contrary, the impact of nitrogen varied according to the inoculation modality, with a negative effect on the production of glycerol during *S. cerevisiae* pure culture, and conversely, increasing the formation of glycerol when *M. pulcherrima* was inoculated first. This difference may be partly attributable to the specific response of *M. pulcherrima* to an increased nitrogen concentration in terms of glycerol production, which was higher than that of *S. cerevisiae* (Supplementary Data S3). Another reason may be the interactions that occurred between the two yeast species, particularly the changes triggered by *M. pulcherrima* early growth in the *S. cerevisiae* growth environment, particularly regarding the composition and availability of nitrogen resource. Actually, a modulation of glycerol formation according to the nature of the nitrogen source has been reported (Albers et al., 1996; Chen et al., 2014).

Otherwise, the formation of acetate was strongly affected by the early addition of *M. pulcherrima*, with much lower production during sequential fermentation than during pure culture. This decrease was dependent on nitrogen availability, as it was less pronounced when nitrogen was limiting but still substantial. The poor ability of *M. pulcherrima* to produce acetate, which was positively modulated by the nitrogen concentration, was not sufficient to explain the drastic reduction in acetate formation during sequential fermentation, again highlighting the key role of interactions between the species.

### The Impact of Nutrient Availability on the Production of Volatile Compounds Depends on the Inoculation Strategy

The major consequences of early *M. pulcherrima* inoculation before *S. cerevisiae* fermentation on the aroma profile and on its modulation by nutrient availability were associated with the low capacity of this NS strain to produce acetate. This limited capacity likely resulted in the limitation of cytosolic acetyl-CoA, which is mainly synthetized from acetate by acetyl-CoA synthase during fermentation (van Roermund et al., 1995). This explains in turn the low production of medium-chain fatty acids and their ethyl ester derivatives from the precursor acetyl-CoA that we observed under sequential fermentation. For both inoculation modalities, the production of ethyl esters and fatty acids was positively impacted by nitrogen, in agreement with previous studies on *S. cerevisiae* pure culture (Garde-Cerdán and Ancín-Azpilicueta, 2008; Torrea et al., 2011; Rollero et al., 2014) and negatively controlled by lipid availability. This can be explained by the interconnection of the two types of metabolism, as nitrogen and lipids impact the regulation of *ATF1* (Swiegers et al., 2005; Saerens et al., 2008).

Early inoculation with *M. pulcherrima* has a limited impact on the formation of higher alcohols and acetate esters during fermentation and the variations of these products in response to changes in nutrient availability were close to those previously described for *S. cerevisiae* pure culture (Mouret et al., 2014; Rollero et al., 2014). The slight increase generally observed under sequential fermentation may be related to positive interactions between the two species. In particular, the production of higher alcohols and acetate esters by *S. cerevisiae*, which is favored at low to moderate YAN levels (Carrau et al., 2008, 2015; Rollero et al., 2014), may be promoted by changes in the composition of the nitrogen resource due to *M. pulcherrima* consumption. However, some exceptions deserve to be highlighted. First, while a substantial increase in phenylethanol formation associated with the nitrogen content was shown for during *S. cerevisiae* pure culture, no significant effect of nitrogen was observed on the final production of phenylethyl ethanol during *M. pulcherrima*/*S. cerevisiae* sequential fermentation. This observation may be related to the high contribution of *M. pulcherrima* in the production of this compound, which was approximately 40 mg/L after 48 h, regardless of the nitrogen concentration in the medium. Another important difference was related to the production of isobutanol and, to a lesser extent, isobutyl acetate, which was substantially higher under sequential fermentation than in pure culture as a consequence of the high capacity of *M. pulcherrima* to produce isobutanol (Comitini et al., 2011; Sadoudi et al., 2012). This observation may be explained by differences in the management of the α-ketoisovalerate (KIV) pool between the two species, with a lower flux toward α-ketoisocaproate (KIC) being observed in *M. pulcherrima* compared to *S. cerevisiae* as a result of the limited formation of acetyl-coA, the co-substrate for the conversion of KIV to KIC, in *M. pulcherrima*. Finally, comparison of the production of higher alcohols and acetate esters produced via the reductive branch of the Ehrlich pathway with that of compounds from the oxidative branch (medium-chain fatty acids and ethyl esters) revealed increased formation of reduced compounds during sequential fermentation at the expense of the production of oxidized molecules. The reorientation of the carbon flux between these branches suggests a modification of the NAD^+^/NADH balance under sequential inoculation related to the metabolic specificities of *M. pulcherrima* and its further consequences for *S. cerevisiae* metabolism. Concerning acetate esters, the model indicates a positive effect of nitrogen, a negative effect of lipids and a negative interaction between these two parameters under sequential inoculation. The negative effect of lipids on acetate ester production can be explained by the repression of the expression of the *ATF1* gene by lipids, previously reported in *S. cerevisiae* (Fujii et al., 1997; Fujiwara et al., 1999), while *ATF1* overexpression is induced by nitrogen (Verstrepen et al., 2003).

### Varietal Thiol Production Is Increased Under Sequential Inoculation

Recent studies have highlighted the potential of NS species to improve the varietal aroma contents of wines, including that of polyfunctional thiols (Anfang et al., 2009; Zott et al., 2011; Renault et al., 2016; Ruiz et al., 2018). Our work revealed a pronounced effect of early inoculation with *M. pulcherrima* on the production of these mercaptans, increasing the formation of 3MH and 4MMP by a factor of 2 to 9 and leading to the formation of 3MHA in detectable amounts. The differences in the levels of thiol production between sequential and pure culture were striking. As an example, while the formation of 3MH by *S. cerevisiae* in pure culture varied from 72 to 152 ng/L depending on the culture conditions, the range of 3MH production during sequential fermentation was between 213 and 821 ng/L. However, this molecule was not detected after the first 48 h of fermentation with *M. pulcherrima*. This may be related to the low β-lyase activity measured with this strain, in agreement with data reported in literature for other *M. pulcherrima* strains (Zott et al., 2011; Belda et al., 2016). Consequently, the high thiol release observed when *M. pulcherrima* was inoculated prior to *S. cerevisiae* did not result from cumulative production by the two species but reflected a positive interaction between them. Similar overproduction of these molecules has been previously reported during *Torulaspora delbrueckii*/*S. cerevisiae* sequential fermentation (Renault et al., 2016). In this case, the synergetic interaction was explained by the specificities of the two species regarding the degradation of thiol precursors: *T. delbrueckii* only assimilated glutathionylated conjugates, whereas *S. cerevisiae* preferentially converted cysteinylated precursors (Winter et al., 2011). Thus, the efficient degradation of glutathionylated precursors by *T. delbrueckii* increased the availability of cysteinylated precursors, which were then further hydrolyzed to thiols by *S. cerevisiae*. A comparable mechanism might occur during *M. pulcherrima*/*S. cerevisiae* sequential fermentation, but this assumption requires experimental confirmation.

These results differed from reports in the literature that sequential inoculation with *M. pulcherrima* mostly resulted in a decrease in the final concentrations of varietal thiols, except for that of 4MMP in some conditions (Sadoudi et al., 2012; Ruiz et al., 2018). These differences could be explained by the strains used and their variable capacity to release varietal thiols (Ruiz et al., 2018). Interestingly, higher variability in the final concentration of thiols according to nutrient availability was observed under sequential inoculation than in pure culture. Under sequential inoculation, the final concentrations of 3MH and 4MMP were mainly affected by the nitrogen content of must, with a quadratic effect, showing a maximum at 200 mg/L of initial YAN. Different phenomena could explain the effect of nitrogen. At initial YAN concentrations of up to 200 mg/L, the remaining nitrogen concentration was limiting when *S. cerevisiae* was added, which may lead *S. cerevisiae* to hydrolyze more thiol precursors to retrieve amino acids. Thus, a recent study investigating the impact of the must composition on the release of thiols suggested that thiol precursors could be hydrolyzed to alleviate the lack of some amino acids (Alegre et al., 2017). At higher initial nitrogen concentrations, the limitation of thiol release was most likely modulated by nitrogen catabolic repression (NCR), which was active in the presence of preferential nitrogen sources and repressed the synthesis of specific permeases of non-preferred sources. However, the NCR target genes have not yet been clearly identified, which are involved in either the transport of precursors into the cell (Subileau et al., 2008) or the transcription of β-lyases (Thibon et al., 2008). Furthermore, we observed a correlation between the concentrations of 3MH and 4MMP and β-lyase activity (Supplementary Data S8). This activity is therefore regulated by nitrogen availability in some way.

The production of 3MHA was also variable depending on nutrient availability under both inoculation strategies. A positive impact of nitrogen, a negative effect of lipids – in agreement with the negative effect of linoleic acids on 3MHA reported by Casu et al. (2016) – and an interaction between nitrogen and lipids were observed. The profile of 3MHA production depending on nutrient availability was close to that reported for acetate esters (Rollero et al., 2014). This is in agreement with the molecular basis of the formation of this molecule from 3MH, which involves Atf1p (Swiegers et al., 2007), which is also responsible for acetate ester production and repressed by unsaturated fatty acids (Fujii et al., 1997; Verstrepen et al., 2003).

## Conclusion

This work was conducted with two main objectives: the comparison of pure cultures of S. *cerevisiae* and sequential inoculation with *M. pulcherrima*; and the assessment of how nutrient availability impacts fermentation progress and results under these two inoculation strategies. Even if the fermentation time was slightly extended, early inoculation with *M. pulcherrima* had a clear impact on fermentation, characterized by a positive impact on the production of aroma compounds, especially varietal thiols, and reduction in acetate, ethanol and acid production. These differences result from metabolic interactions between the two species, which can be attributed, at least in part, to regulations in *S. cerevisiae* related to *M. pulcherrima* growth-induced environmental changes (nutrient consumption, production of metabolites…). However, the metabolic and molecular basis governing these interactions are still unclear and will necessitate thorough studies. The management of must parameters appears to be crucial, especially regarding the initial concentrations of nitrogen and lipids. As *M. pulcherrima* consumes nitrogen in the first stage of fermentation, winemakers need to be aware of the risk of nitrogen deficiencies for *S. cerevisiae*, which can lead to sluggish fermentation. Otherwise, different optimum nutrient concentrations were obtained depending on the target aroma compound. Indeed, the maximal production of thiols was obtained with 200 mg/L of YAN and 8 mg/L of lipids, while the maximal concentration of acetate esters was obtained with 300 m/L of nitrogen and 2 mg/L of lipids. Consequently, a compromise must be found in terms of yeast nutrition, to promote the formation of targeted aromas while simultaneously ensuring the completion of fermentation.

## Data Availability Statement

All datasets generated for this study are included in the article/Supplementary Material.

## Author Contributions

CC, PS, and AO-J conceived and designed the study and contributed to the statistical analysis and interpretation of the data. PS implemented the experiment and drafted the manuscript. CC revised the manuscript. All authors read and approved the final version of the manuscript.

## Conflict of Interest

PS and AO-J were employed by the Lallemand.

The remaining author declares that the research was conducted in the absence of any commercial or financial relationships that could be construed as a potential conflict of interest.
