# Supplementary material for: Impact of Nutrient Availability on the Fermentation and Production of Aroma Compounds Under Sequential Inoculation With M. pulcherrima and S. cerevisiae
Source: Front Microbiol. 2020 Feb 28;11:305. doi: 10.3389/fmicb.2020.00305 (PMC7058555; doi:10.3389/fmicb.2020.00305)
Supplement: Supplementary file 1 [file Data_Sheet_1.pdf]

Supplementary Datasheets S1-S8

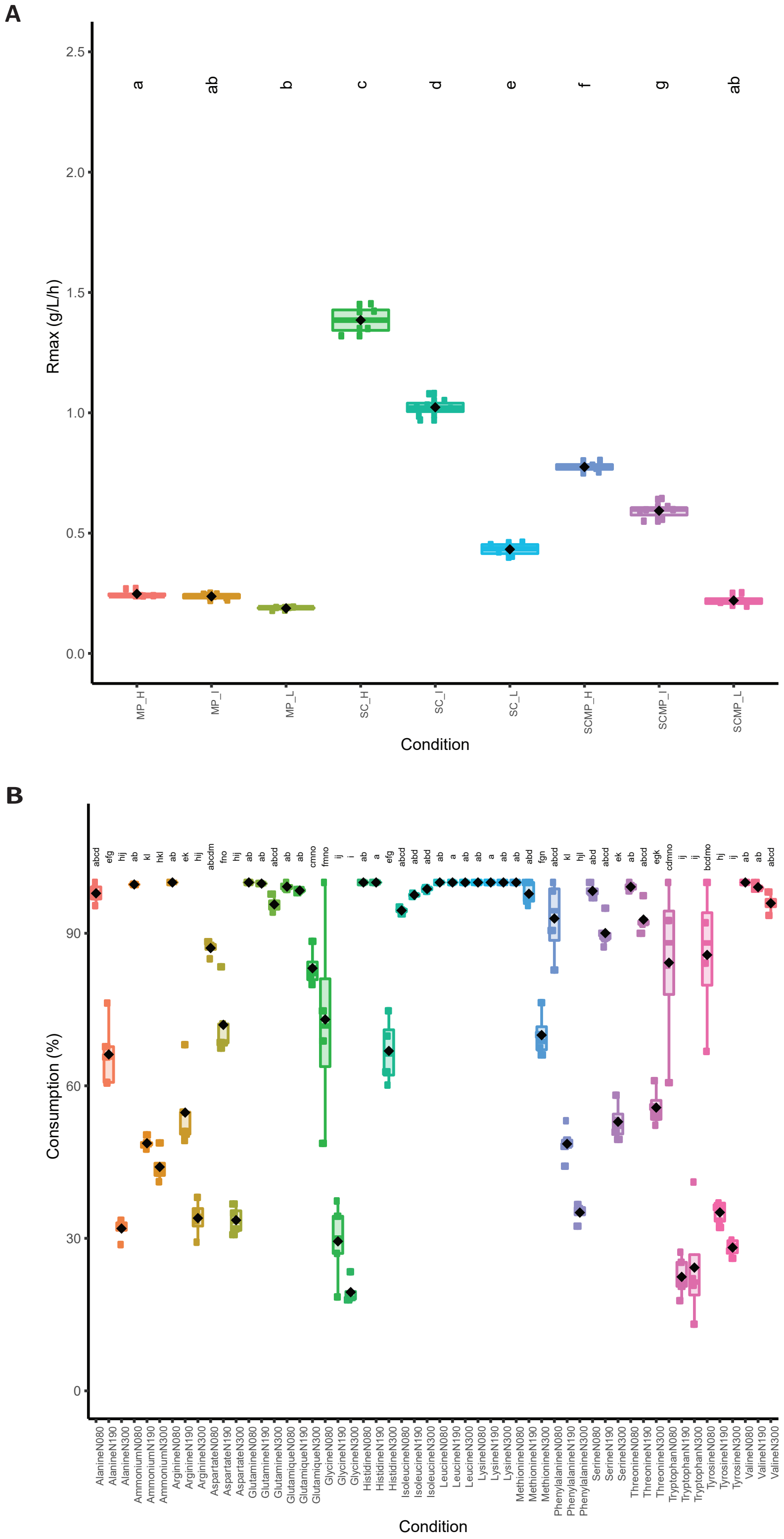

Supplementary data 1: A. Box-plots of the fermentation rate Rmax depending on nitrogen availability (Low, Intermediate, High) measured during S. cerevisiae pure cultures (SC), during the first 48 h (MP) or after S. cerevisiae inoculation (SCMP) during sequential fermentation.
B. Box-plots of the consumption of nitrogen sources (expressed in percentage of the initial concentration) after 48 h of M. pulcherrima fermentation depending on the nitrogen availability (low, intermediate, high). Means sharing a letter are not significantly different (Wilcoxon pairwise comparison test, Holm p-value adjustment method).

Supplementary data 2: Amino acid composition of the medium in mg N/L after 48 h of fermentation with *M. pulcherrima*. Conditions are labelled as sugar-nitrogen-lipids.

	180-80-5	220-80-2	220-80-8	260-80-5	180-190-2	180-190-8	220-190-5a	220-190-5b	220-190-5c	260-190-2	260-190-8	180-300-5	220-300-2	220-300-8	260-300-5
Proline	10.82	11.21	12.20	11.86	29.46	32.54	30.31	30.30	30.68	28.16	29.85	47.31	46.78	45.02	45.18
Alanine	0.00	0.13	0.05	0.21	2.63	4.36	4.37	3.79	4.11	3.58	3.80	11.62	12.46	11.74	11.80
Arginine	0.00	0.00	0.00	0.00	12.48	17.67	19.85	18.66	19.13	19.15	19.36	38.25	43.69	40.06	41.09
Aspartate	0.09	0.10	0.10	0.12	0.31	0.52	0.61	0.55	0.60	0.59	0.59	1.88	2.06	1.93	2.02
Glutamine	0.00	0.00	0.00	0.00	0.09	0.09	0.10	0.09	0.10	0.09	0.09	1.49	3.32	2.64	3.80
Glutamate	0.00	0.03	0.02	0.03	0.08	0.11	0.08	0.09	0.09	0.08	0.09	1.01	1.65	1.49	1.76
Glycine	0.00	0.22	0.18	0.36	1.06	1.37	1.23	1.23	1.21	1.11	1.18	2.18	2.18	2.17	2.04
Histidine	0.00	0.00	0.00	0.00	0.00	0.00	0.00	0.00	0.00	0.00	0.00	0.66	0.88	0.55	0.82
Isoleucine	0.04	0.04	0.04	0.04	0.04	0.05	0.04	0.04	0.04	0.05	0.04	0.04	0.03	0.03	0.05
Leucine	0.00	0.00	0.00	0.00	0.00	0.00	0.00	0.00	0.00	0.00	0.00	0.00	0.00	0.00	0.00
Lysine	0.00	0.00	0.00	0.00	0.00	0.00	0.00	0.00	0.00	0.00	0.00	0.00	0.00	0.00	0.00
Methionine	0.00	0.00	0.00	0.00	0.00	0.00	0.06	0.02	0.04	0.04	0.05	0.48	0.69	0.61	0.67
Phenylalanine	0.00	0.06	0.01	0.11	0.74	0.80	0.88	0.86	0.86	0.82	0.82	1.60	1.69	1.62	1.58
Serine	0.00	0.04	0.03	0.06	0.25	0.55	0.63	0.47	0.55	0.49	0.54	3.27	3.95	3.65	3.83
Threonine	0.00	0.02	0.01	0.03	0.11	0.36	0.44	0.30	0.36	0.33	0.36	2.71	3.32	3.07	3.22
Tryptophan	0.00	0.45	0.21	1.09	4.78	5.17	5.40	5.28	5.30	5.22	4.91	8.22	9.01	6.11	8.09
Tyrosine	0.00	0.04	0.02	0.09	0.42	0.46	0.45	0.45	0.45	0.43	0.42	0.77	0.78	0.75	0.74
Valine	0.00	0.00	0.00	0.00	0.00	0.03	0.03	0.03	0.03	0.03	0.04	0.08	0.16	0.18	0.27
NH4	0.00	0.00	0.00	0.00	42.53	39.47	37.22	37.94	40.86	41.07	40.78	55.71	56.87	56.87	55.13
Total	10.95	12.35	12.90	14.02	95.12	103.54	101.81	100.20	104.52	101.37	103.08	177.43	189.64	178.65	182.22

Supplementary data 3: Biomass concentration in cells/mL at different times of fermentation under sequential inoculation or in pure cultures of *S. cerevisiae*. MP: *M. pulcherrima*, SC: *S. cerevisiae*. 90%: 90% of fermentation progress. Conditions are labelled as sugar-nitrogen-lipids.

	Sequential inoculation						Pure culture SC
	48h		96h		90%		90%
	MP	SC	MP	SC	MP	SC	SC
180-80-5	6.94E+07	0.00E+00	5.91E+07	2.32E+07	0.00E+00	9.09E+07	9.11E+07
220-80-2	6.13E+07	0.00E+00	5.72E+07	1.40E+07	0.00E+00	8.54E+07	8.35E+07
220-80-8	6.50E+07	0.00E+00	6.08E+07	2.06E+07	0.00E+00	8.27E+07	7.94E+07
260-80-5	6.13E+07	0.00E+00	5.76E+07	1.57E+07	0.00E+00	8.19E+07	7.18E+07
180-190-2	7.64E+07	0.00E+00	5.92E+07	4.19E+07	0.00E+00	1.18E+08	1.66E+08
180-190-8	6.82E+07	0.00E+00	9.56E+07	5.24E+07	0.00E+00	1.21E+08	1.63E+08
220-190-5a	6.74E+07	0.00E+00	5.93E+07	4.02E+07	0.00E+00	1.20E+08	1.57E+08
220-190-5b	6.93E+07	0.00E+00	8.68E+07	4.66E+07	0.00E+00	1.25E+08	1.37E+08
220-190-5c	7.15E+07	0.00E+00	6.00E+07	6.34E+07	0.00E+00	1.24E+08	1.47E+08
260-190-2	7.23E+07	0.00E+00	6.23E+07	5.35E+07	0.00E+00	1.11E+08	1.26E+08
260-190-8	7.00E+07	0.00E+00	8.45E+07	4.99E+07	0.00E+00	1.13E+08	1.38E+08
180-300-5	8.25E+07	0.00E+00	8.15E+07	7.90E+07	8.69E+06	1.24E+08	1.77E+08
220-300-2	7.90E+07	0.00E+00	6.38E+07	6.71E+07	1.67E+06	1.49E+08	1.85E+08
220-300-8	6.36E+07	0.00E+00	5.85E+07	8.87E+07	0.00E+00	1.47E+08	1.15E+08
260-300-5	6.59E+07	0.00E+00	6.77E+07	6.54E+07	0.00E+00	1.32E+08	1.82E+08

Supplementary data 4: Concentrations in g/L of ethanol, glycerol, succinate and acetate at 90% of fermentation progress under sequential inoculation and in pure cultures of *S. cerevisiae*. Conditions are labelled as sugar-nitrogen-lipids.

	Sequential fermentation				Pure culture SC			
	Ethanol	Glycerol	Succinate	Acetate	Ethanol	Glycerol	Succinate	Acetate
180-80-5	75.9	7.9	1.85	0.28	81.9	6.2	1.29	0.27
220-80-2	91.2	9.5	1.95	0.34	88.5	6.4	1.22	0.42
220-80-8	94.1	9.4	1.91	0.31	98.5	7.1	1.41	0.43
260-80-5	107.4	11.3	2.10	0.44	107.8	7.8	1.49	0.63
180-190-2	78.5	8.7	1.75	0.01	84.3	5.6	0.88	0.35
180-190-8	75.8	8.8	1.92	0.01	85.3	5.9	0.97	0.36
220-190-5a	83.9	9.2	1.80	0.02	100.7	6.5	1.07	0.50
220-190-5b	90.2	9.6	1.93	0.02	99.5	6.3	1.04	0.49
220-190-5c	91.0	9.6	1.94	0.02	100.0	6.5	1.07	0.49
260-190-2	109.5	10.6	1.92	0.06	113.6	6.9	1.35	0.65
260-190-8	109.3	11.1	2.12	0.02	123.5	8.0	1.41	0.69
180-300-5	81.7	9.5	1.77	0.00	60.1	4.8	0.64	0.27
220-300-2	95.4	9.6	1.51	0.05	95.9	5.8	0.96	0.43
220-300-8	91.8	10.3	1.82	0.01	102.2	6.4	0.90	0.46
260-300-5	106.8	11.0	1.74	0.04	118.0	7.1	2.46	0.64

Supplementary data 5: Concentrations in mg/L of aromas at 90% of fermentation progress under sequential inoculation. Conditions are labelled as sugar-nitrogen-lipids.

	180-80-5	220-80-2	220-80-8	260-80-5	180-190-2	180-190-8	220-190-5a	220-190-5b	220-190-5c	260-190-2	260-190-8	180-300-5	220-300-2	220-300-8	260-300-5
Propanol	9.76	10.54	8.38	13.89	17.42	15.29	17.05	10.66	28.77	20.17	16.87	28.79	31.86	36.94	38.77
Isobutyl alcohol	146.48	115.07	125.62	123.07	135.79	153.59	147.86	99.54	168.66	146.50	136.31	125.67	118.46	153.63	152.13
Isoamyl alcohol	53.94	51.98	54.07	62.75	68.57	66.21	76.88	54.98	86.91	89.69	76.38	52.36	47.04	66.18	68.69
Phenylethyl alcohol	151.90	96.46	88.82	96.42	114.27	105.75	116.51	84.65	131.01	101.82	82.69	125.72	103.08	99.03	83.83
Isobutyl acetate	226.81	536.23	538.47	571.44	321.18	606.84	835.08	815.78	758.49	757.37	1209.37	1661.92	4184.77	1410.75	2151.14
Isoamyl acetate	0.27	0.38	0.31	0.51	0.76	0.53	0.95	0.71	1.12	1.87	1.06	1.60	3.94	1.65	3.24
Phenylethyl acetate	0.69	0.62	0.75	0.87	0.96	0.77	1.71	1.24	2.35	2.42	1.11	2.67	7.87	3.02	6.73
Ethyl isobutyrate	0.02	0.02	0.02	0.03	0.02	0.02	0.02	0.02	0.02	0.02	0.02	0.02	0.02	0.02	0.02
Ethyl hexanoate	0.13	0.17	0.12	0.17	0.32	0.31	0.43	0.29	0.40	0.58	0.45	0.42	0.68	0.45	0.63
Ethyl octanoate	0.09	0.10	0.09	0.12	0.15	0.23	0.31	0.17	0.21	0.31	0.30	0.25	0.42	0.28	0.48
Ethyl decanoate	0.11	0.10	0.11	0.12	0.13	0.30	0.36	0.25	0.23	0.33	0.39	0.27	0.41	0.42	0.62
Isobutyric acid	1.36	1.32	1.56	1.41	1.04	1.24	1.09	0.78	1.02	1.08	1.09	0.94	0.88	0.94	0.86
Hexanoic acid	0.41	0.49	0.46	0.48	1.07	0.89	0.94	0.86	0.89	1.01	0.83	1.05	1.68	0.92	1.12
Octanoic acid	1.37	1.56	1.41	1.55	1.87	1.99	2.26	2.03	2.01	2.60	2.54	2.36	3.93	2.13	2.74
Decanoic acid	0.29	0.59	0.50	0.68	0.97	1.01	1.50	1.67	1.02	2.41	2.94	0.86	2.38	1.15	2.01

Supplementary data 6: Concentrations in mg/L of aromas at 90% of fermentation progress in pure culture of *S. cerevisiae*. Conditions are labelled as sugar-nitrogen-lipids.

	180-80-5	220-80-2	220-80-8	260-80-5	180-190-2	180-190-8	220-190-5a	220-190-5b	220-190-5c	260-190-2	260-190-8	180-300-5	220-300-2	220-300-8	260-300-5
Propanol	14.21	11.03	15.59	11.82	17.12	15.61	21.74	16.44	15.92	25.42	18.99	26.21	31.77	31.25	26.82
Isobutyl alcohol	29.09	27.43	39.73	40.95	30.48	39.38	53.64	38.86	38.88	56.40	62.15	25.31	31.84	49.28	40.14
Isoamyl alcohol	48.29	44.58	60.73	56.33	56.96	56.20	84.69	60.92	60.44	70.69	63.37	38.75	49.68	50.64	47.06
Phenylethyl alcohol	53.51	53.67	54.86	56.86	102.88	104.81	134.31	117.16	95.73	124.16	105.60	89.81	107.88	100.10	95.09
Isobutyl acetate	213.30	194.28	197.14	301.05	173.65	342.42	69.59	213.51	398.34	94.69	526.67	480.84	339.12	887.23	926.67
Isoamyl acetate	0.31	0.32	0.36	0.39	0.69	0.54	0.70	0.73	0.77	0.81	0.88	1.33	1.75	1.69	1.86
Phenylethyl acetate	0.69	0.52	0.70	1.09	1.20	2.00	2.43	2.33	1.64	2.02	2.35	3.50	4.11	3.43	3.50
Ethyl isobutyrate	0.03	0.02	0.03	0.04	0.02	0.02	0.02	0.02	0.02	0.02	0.03	0.00	0.00	0.02	0.02
Ethyl hexanoate	0.40	0.47	0.41	0.39	0.82	0.45	0.47	0.54	0.56	0.73	0.44	0.53	0.83	0.51	0.53
Ethyl octanoate	0.31	0.30	0.40	0.35	0.76	0.37	0.44	0.50	0.64	0.85	0.45	0.50	0.86	0.68	0.63
Ethyl decanoate	0.31	0.24	0.53	0.36	0.97	0.61	0.83	0.88	1.18	1.00	0.73	1.00	1.42	1.47	1.11
Isobutyric acid	3.46	3.03	4.61	5.48	1.85	2.01	2.65	2.28	2.40	3.50	4.26	1.16	1.38	1.70	1.92
Hexanoic acid	1.40	1.52	1.36	1.28	2.39	1.70	1.12	1.75	1.82	1.70	1.46	2.28	2.58	1.79	2.19
Octanoic acid	2.95	3.67	3.28	3.39	5.32	3.88	2.10	3.81	4.74	3.39	4.32	4.69	5.20	5.16	5.56
Decanoic acid	3.36	4.21	5.06	5.12	5.93	5.28	1.72	5.44	7.78	8.31	8.05	6.09	5.11	8.63	8.36

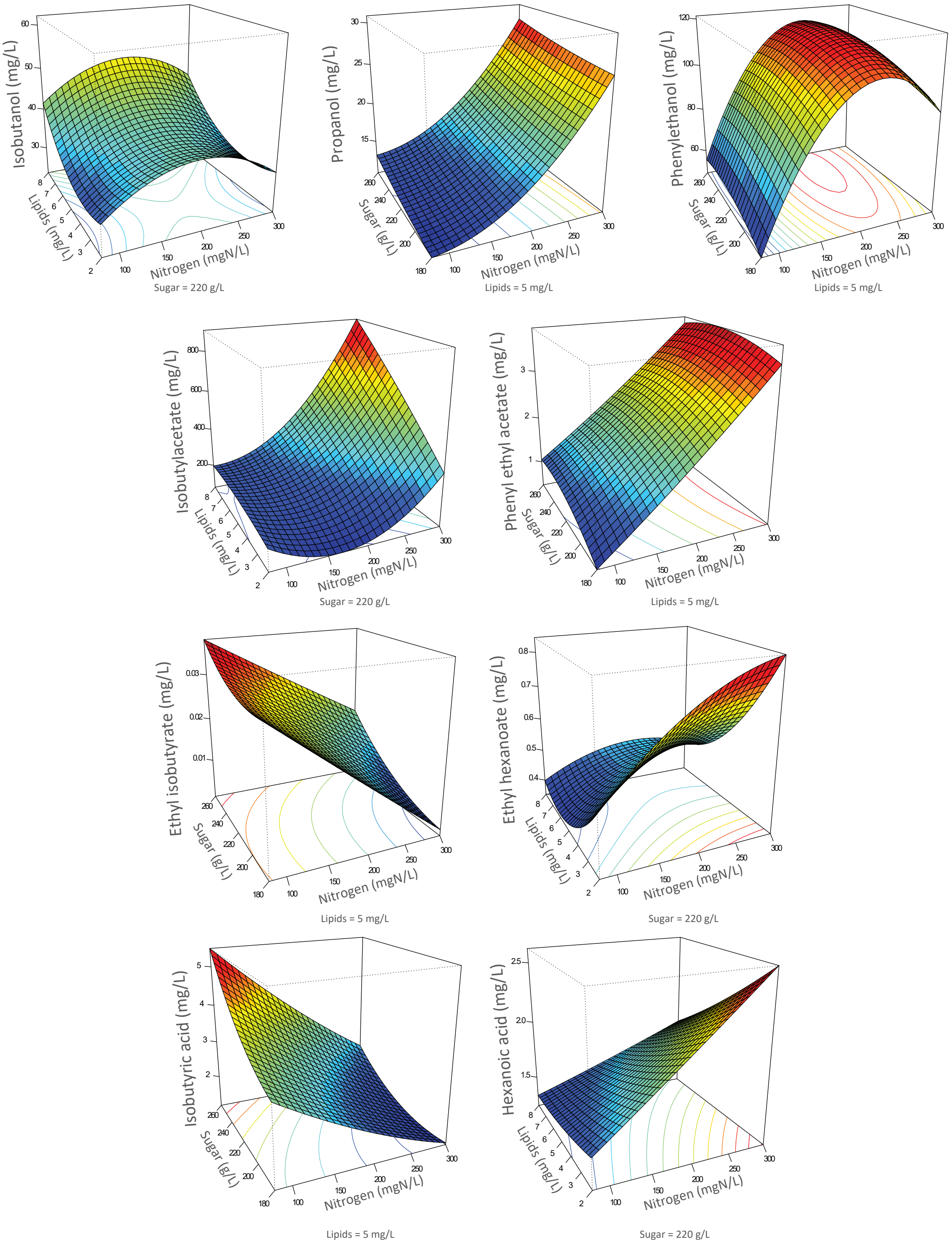

Supplementary data 7: Response surfaces of fermentative aromas in relation to the variation of sugar, lipids and nitrogen in pure cultures of S. cerevisiae. For all graphs, one parameter is fixed.

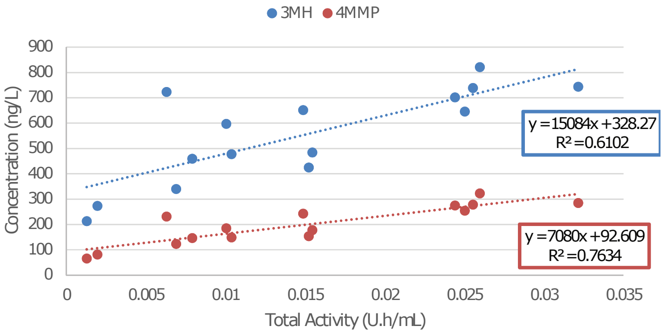

Supplementary data 8: Correlation between 3MH (blue dots) or 4MMP (red dots) and the total β-lyase activity measured during sequential fermentation.
